# Detailed Analysis of Temporal Features on Contrast Enhanced Ultrasound May Help Differentiate Intrahepatic Cholangiocarcinoma from Hepatocellular Carcinoma in Cirrhosis

**DOI:** 10.1371/journal.pone.0098612

**Published:** 2014-05-29

**Authors:** Rui Li, Meng-Xia Yuan, Kuan-sheng Ma, Xiao-Wu Li, Chun-Lin Tang, Xiao-Hang Zhang, De-Yu Guo, Xiao-Chu Yan

**Affiliations:** 1 Department of Ultrasound, Southwest Hospital Affiliated to Third Military Medical University, Chongqing, China; 2 Department of Hepato-biliary-Pancreatic Surgery, Southwest Hospital Affiliated to Third Military Medical University, Chongqing, China; 3 Department of Pathology, Southwest Hospital Affiliated to Third Military Medical University, Chongqing, China; Kaohsiung Medical University Hospital, Kaohsiung Medical University, Taiwan

## Abstract

**Aim:**

To verify if detailed analysis of temporal enhancement patterns on contrast enhanced ultrasound (CEUS) may help differentiate intrahepatic cholangiocarcinoma (ICC) from hepatocellular carcinoma (HCC) in cirrhosis.

**Methods:**

Thirty three ICC and fifty HCC in cirrhosis were enrolled in this study. The contrast kinetics of ICC and HCC was analyzed and compared.

**Results:**

Statistical analysis did not reveal significant difference between ICC and HCC in the time of contrast first appearance and arterial peak maximum time. ICC displayed much earlier washout than that of HCC (47.93±26.45 seconds vs 90.86±31.26 seconds) in the portal phase, and most ICC (87.9%) showed washout before 60 seconds than HCC (16.0%). Much more ICC (78.8%) revealed marked washout than HCC (12.0%) while most HCC (88.0%) showed mild washout or no washout in late part of the portal phase (90–120 seconds). Twenty six out of thirty three ICC (78.8%) demonstrated both early washout(<60seconds) and marked washout in late part of the portal phase, whereas, only six of fifty HCC (12.0%)showed these temporal enhancement features (*p* = 0.000).When both early washout and marked washout in the portal phase are taken as diagnostic criterion for ICC, the diagnostic sensitivity, specificity, positive predictive value, negative predictive value and accuracy were 78.8%,88.0%,81.3%,86.3%,and 84.3% respectively by CEUS.

**Conclusions:**

Analysis of detailed temporal enhancement features on CEUS is helpful differentiate ICC from HCC in cirrhosis.If a nodule in cirrhotic liver displays hyper-enhancement in the arterial phase followed by early and marked washout in the portal phase, the nodule is highly suspicious of ICC rather than HCC.

## Introduction

Intrahepatic peripheral cholangiocarcirnoma (ICC) constitutes the second most common primary liver malignant tumor in cirrhotic patients, accounting for 1%–3% of newly developed tumors[1], [2],and the incidence of ICC appears raising,especially in Western countries [3]. Cirrhotic patients may undergo surveillance for hepatocellular carcinoma (HCC) by ultrasound every 3–6 months [4], [5].When a focal liver lesion is detected by ultrasound in a cirrhotic patient, a high suspicion of HCC arises. However, not all malignant tumors in cirrhosis are HCC, theoretically, ICC also may be found in cirrhosis. Differentiation between them is essential for surgical planning and prognosis assessment because ICC and HCC have different biological behaviour and prognosis[6], [7].The European Federation of Societies for Ultrasound in Medicine and Biology (EFSUMB)defined typical contrast enhanced ultrasound (CEUS) vascular pattern of ICC as rim-like enhancement/non-enhancement in arterial phase followed by hypo/non-enhancement during the portal and late phases [8], but these recommendations are based on studies in which most patients did not have underlying cirrhosis. There is little information regarding the CEUS pattern of ICC in cirrhosis. Up to now, only three retrospective studies including a relatively limited number of patients have described the ICC enhancement pattern at CEUS and the associated risk of misdiagnosis with HCC [9], [10], [11]. These studies showed that CEUS was at risk of misdiagnosis of ICC for HCC in about 47.6%-68.8% patients. However, some CEUS features may help suspect ICC, especially in some cases with inconclusive CT or MRI [11], [12] and this topic remains of high interest. Hence, the principle aim of our study was to compare CEUS imaging features of ICC and HCC in cirrhotic patients, to verify if detailed analysis of temporal enhancement patterns on CEUS may help differentiate ICC from HCC in this compelling clinical situation.

## Methods

### Patients

We retrospectively analyzed patients who were diagnosed with ICC and HCC on cirrhosis in which CEUS had been performed in our hospital between January 2005 and February 2014.The following inclusion criteria were applied:

- Histologically proven liver cirrhosis by representative resections or biopsies from the liver parenchyma;
- The diagnosis of ICC and HCC had to be pathologically proven (through assessment of surgical specimen or biopsy);
- Real-time CEUS was performed for diagnosis within less than a month before surgical operation or biopsy

Exclusion criteria were:

- Patients with mixed hepatocellular-cholangiocellular carcinoma.
- Systemic chemotherapy or targeted therapy prior to the CEUS.
- ICC and HCC without cirrhosis.

HCC sample cases were randomly selected to form the study group by using the sequence numbers created by the SPSS 13.0 software package (SPSS Inc, Chicago, IL).

The study protocol and consent procedure of the patients were approved by the institutional ethics board of Southwest Hospital. All patients signed their written informed consent to participate in this study after thorough explanation of the study protocol and procedure. Written informed consent according to the ethical guidelines from Declaration of Helsinki was obtained from each patient in this study. We performed a per-patient analysis. In a patient with more than one solid lesion in the liver, only the largest one was measured and investigated because CEUS could not scan multiple nodules simultaneously after one injection of contrast agent if the nodules are not at the same scan plane.

### Image acquisition

US examinations were performed by two experienced physicians with over 15 years of experience of liver ultrasound examination (R L, and XH Z) with an Acuson Sequoia 512 ultrasound unit (Siemens Medical Solutions, Santa Clara, Calif). Baseline US was performed with a multi-frequency 4C1 convex array probe. The gray-scale US characteristics of the lesion, including location, size, shape, and echogenicity were recorded. CEUS was performed by using contrast pulse sequencing (CPS) imaging. Split screen image was used to show the same scanning plane of gray scale ultrasound simultaneously. Real-time contrast imaging setting was used with a low mechanical index of <0.2 to avoid the microbubbles disruption. A volume of 2.4 mL of a second-generation US blood pool contrast agent (SonoVue, Bracco Imaging B.V, Geneva, Switzerland) was injected into cubital vein in bolus via a 20-gauge needle followed by a 5 mL saline flush. After contrast medium injection, hepatic lesion was scanned continuously for up to 4 min. The whole vascular phase was studied, consisting of the arterial phase (0–30 s from beginning of contrast agent bolus injection), portal phase (31–120 s after the injection), and delayed phase (121–240 s after the injection) according to EFSUMB recommendations [8]. In each case, the enhancing intensity and pattern of the target nodule during each vascular phase were recorded. For specified temporal analysis, the portal phase was divided into three parts, namely early part (31–60 s from beginning of contrast agent bolus injection), middle part (61–90 s after the injection) and late part (91–120 s after the injection) of the portal phase.

### CEUS image analysis

The contrast vascular patterns on CEUS were defined by comparing the enhancement behaviour of the tumor with the surrounding liver parenchyma and were classified as:

1. Peripheral hyper-enhancement—irregular rim-like hyper-enhancement at the peripheral part of the lesion with sparse filiform and punctiform internal enhancement.
2. Heterogeneous hyper-enhancement—when the lesion displays mixed hyper-enhancement inhomogeneously at both the periphery and the central part of the lesion.
3. homogeneous hyper-enhancement—when the whole lesion shows hyper-enhancement homogeneously.
4. Hypo-enhancement—the lesion enhances in the less degree than that of the surrounding liver parenchyma.
5. Iso-enhancement—the lesion enhances in the similar degree as the surrounding liver parenchyma.
6. Non-enhancement—no appearance of contrast agent (microbubbles) at both the periphery and the central part of the lesion.

Wash-out appearance was considered as the presence of hypo-enhancement of the lesion in the portal or late phases preceded by hyper-enhancement in the arterial phase, a lesion lacking any enhancement in all phases was not defined as washout. When a nodule shows heterogeneous hyper-enhancement in the arterial phase, observation of washout was confined to the area showing hyper-enhancement in the arterial phase, and the area showing non-enhancement was excluded from the observation. The extent of washout was classified as:

1. Marked washout—when the lesion displays obviously lower echogenicity than the surrounding liver parenchyma in the portal or late phases preceded by hyper-enhancement in the arterial phase.
2. Mild washout —when the lesion shows slightly lower echogenicity than the surrounding liver parenchyma in the portal or late phases preceded by hyper-enhancement in the arterial phase.
3. No washout—when the lesion enhances in the similar degree or higher degree as the surrounding liver parenchyma in the portal or late phases preceded by hyper-enhancement in the arterial phase.

The tumor contrast kinetics was analyzed by review on the recorded clips. The time of contrast first appearance, the time of peak maximum enhancement in the arterial phase, time of emergence of washout, in comparison to the surrounding liver tissue was documented. Intensity of peak maximum enhancement of tumor in the arterial phase, intensity of tumor enhancement in late part of the portal phase (still frames from the CEUS cine clips 90–120 seconds after contrast agent injection) and intensity of tumor enhancement of the late phase (still frames from the CEUS cine clips 170–190 seconds after contrast agent injection)were measured suing a Sonomath software (AMBITION BIOTRCH, Chongqing, China).A circular region of interest (ROI) with a size corresponding to that of the tumor and another ROI in the adjacent non-tumor liver parenchyma with similar depth were manually drawn.Tumor/tissue ratio of enhancement intensity was calculated by dividing enhancement intensity of tumor with enhancement intensity of adjacent liver parenchyma in the same still image.

### CT scan

Abdomen CT was performed with multi-detector-row CT (MDCT, Definition, Siemens, Erlangen, Germany) using a 4 phase contras-enhanced protocol (unenhanced, hepatic arterial, portal venous, and delayed phases). First, an unenhanced scan was obtained through the liver. Next, after intravenous infusion of 2 ml/kg of a nonionic iodine-containing contrast agent (ultravist 370, Scherning AG, Berlin, Germany) using a power injector (Stellant CT Injection System, Medrad, Indianola, Pennsylvania) at a rate of 4.0 ml/sec. Contrast-enhanced scans were obtained in arterial with bolus test trigger for optimal characterization of focal hepatic lesions. Data acquisitions were obtained through the whole liver in a craniocaudal direction during a single breath-hold helical acquisition for 4–6 sec with 5 mm slice thickness and 0.5 s rotation time. The acquisition of the arterial phase was automatically started 5 s after contrast agent reaching the threshold in the aorta. The start of acquisition sequences was 60 s for the portal venous phase and 180 s for the delayed phase.

### Categorization of enhancement patterns at CT

The enhancement through each of the different phases after intravenous contrast administration was registered as follows: (1) globally hyperdense: increased signal relative to the surrounding liver parenchyma, involving the totality of the lesion; (2) partially hyperdense: increased signal involving more than 25% of the lesion cross-section area with a non-homogeneous distribution; (3)peripherally hyperdense: increased signal limited to the periphery of the lesion, involving less than 25% of its area, resembling a rim-like pattern; (4) isodense: same density as the surrounding liver parenchyma; (5) hypodense: lower density compared to the liver parenchyma involving more than half of the cross-sectional area of the tumor. Dynamic pattern of enhancement was defined according to the analysis of the progression of contrast enhancement over the progressive different phases of the study, as follows: (1) stable persistent contrast enhancement: the nodule enhancement is unmodified from the arterial to the portal venous and delayed phases; (2) progressive contrast enhancement: the nodule enhances progressively over time, reaching maximal intensity in delayed phases; (3) ‘wash-out’ pattern: global/partial hyperdense of the lesion during the arterial phase followed by hypodense in portal and/or delayed venous phases; (4) all other cases. This classification was adopted from Rimola et al [13]and lavarone et al[14]

CT findings were evaluated in consensus by 2 abdominal radiologists (PC and SYD) with 14 and 22 years of experience in liver radiology who were blinded to the contrast-enhanced ultrasonographic findings and pathological results of the tumors.

### Statistical analysis

We performed a per-patient analysis in this study. Baseline characteristics of the patients are expressed as median and range or count and proportion. Comparison of patients with ICC and patients with HCC was done by using the Student t test for continuous variables and Fisher, s exact test for categorical variables. A *P* value of less than 0.05 was considered statistically significant. Statistical analysis were performed using the SPSS 13.0 software package (SPSS Inc, Chicago, IL)

## Results

### Characteristics of the patients

Due to the inclusion–exclusion criteria of the study,some ICC patients were excluded because they had: (1) ICC without histological evidence for liver cirrhosis (n = 84); (2) mixed hepatocellular-cholangiocellular carcinomain in cirrhotic patients (n = 28).Finally, this retrospective search yielded a total 33 histologically proven ICC in cirrhotic patients. Twenty six cases of ICC were diagnosed histologically after resection (78.8%) and 7 cases after biopsy (21.2%). Some HCC patients were excluded because: (1) No histological evidence for liver cirrhosis (n = 392); (2)Transarterial chemoembolization therapy prior to CEUS (n = 140).Fifty histologically proven HCC (10 well differentiated HCC,25moderately differentiated HCC and 15 poorly differentiated HCC) were randomly selected from 897 HCC in cirrhotic patients to form the HCC study group. Forty eight cases of HCC were diagnosed histologically after resection (96%) and 2 cases after biopsy (4%). The characteristics of the cirrhotic patients with ICC and HCC are presented in Table 1. Patients with ICC and HCC showed no statistical difference in terms of age, sex, etiology, Child-Pugh class and tumor size. Patients with HCC had higher AFP levels than those with ICC.AFP exceeded 200 ng/ml in more HCC patients (38.0%) than that in ICC patients (12.1%, *p* = 0.032).The echogenicity patterns of ICC were similar to that of HCC on conventional ultrasound.

**Table 1:** Demographics of patients with ICC and HCC in cirrhosis.

	ICC	HCC	*P*
Age			
mean (year)	49.21±8.99	53.24±11.39	0.077
Median (year)	32–75, 48	31–76, 53.5	
Male/female (n)	29/4	43/7	0.805
AFP (ng/ml)			
<20	23	23	
20–200	6	8	
>200	4	19	0.032
Child-pugh classification			
A	29	44	0.987
B	4	6	
C	0	0	
Nodule size (mm)			
mean±SD	49.91±27.07	59.14±23.09	0.890
Median(range)	40 (17–114)	42.5 (17–103)	
Number of nodules (n)			
1	27	37	0.640
2	4	8	
3	0	2	
>3	2	3	
Etiology of cirrhosis			
Hepatitis B	28	46	0.305
Hepatitis C	0	0	
Hepatitis B and hepatitis C	0	0	
Others	5	4	
Echogenicity of nodules			
hypoechoic	17	24	0.941
isoechoic	1	1	
hyperechoic	5	10	
mixed	10	15	

### Characteristics of CEUS enhancement

The perfusion characteristics of ICC and HCC on CEUS are demonstrated in Table 2.The parametric measurement of ICC and HCC perfusion features at CEUS are presented in Table 3.

**Table 2:** Perfusion characteristics of ICC and HCC on CEUS in cirrhotic patients.

Characteristics	ICC	HCC	*P*
Arterial phase			
Contrast first appearance (s)			
mean±SD	12.63±4.71	12.46±3.82	0.852
Median(range)	5–24, 11	6–23, 12.5	
Arterial peak maximum (s)			
mean±SD	20.24±5.66	21.04±4.70	0.488
Median(range)	9–33, 20	13–34, 20	
Hyperenhancement			
Homogeneous	10 (30.3%)	24 (48.0%)	0.109
Heterogeneous	23 (69.7%)	26 (52.0%)	
Portal phase			0.000
Mild washout	6 (18.2%)	33 (66.0%)	
Marked washout	26 (78.8%)	6 (12.0%)	
No washout	1 (3.0%)	11 (22.0%)	
Late phase			0.000
Mild washout	3 (9.1%)	27 (54.0%)	
Marked washout	30 (90.9%)	22 (44.0%)	
No washout	0 (0%)	1 (2.0%)	
Washout first appearance (s)			
mean±SD	47.93±26.45	90.86±31.26	0.000
Median(range)	41 (21–153)	83(36–185)	
Emergence of washout			
<60S	29 (87.9%)	8 (16.0%)	0.000
61–120S	3 (9.1%)	36 (72.0%	
>120S	1 (3.0%)	6 (12.0%)	

**Table 3:** Parametric measurements of ICC and HCC perfusion features on CEUS.

	ICC	HCC	*P*
Arterial phase			
Peak intensity of tumors (db)			
Mean±SD	97.29±36.81	121.97±28.77	0.001
Median(range)	101.90 (26.38–182.87)	122.82 (47.31–173.77)	
Tumor/tissue ratio of intensity			
Mean±SD	1.44±0.52	1.86±0.69	0.003
Median(range)	1.33 (0.43–2.69)	1.72 (0.99–3.65)	
Portal phase			
Lowest intensity of tumor(db)			
Mean ±SD	41.71±18.31	66.94±21.34	0.000
Median(range)	41.85 (8.19–105.54)	68.24(20.93–135.56)	
Lowest tumor/tissue ratio of intensity in late portal phase (90–120 s)			
Mean±SD	0.44±0.14	0.77±0.15	0.000
Median(range)	0.42 (0.26–0.94)	0.81 (0.34–0.99)	
Late phase			
Lowest intensity of tumor(db) in late phase (170–190 s)			
Mean±SD	33.42±14.49	51.72±15.69	0.000
Median(range)	32.75(6.37–83.7)	52.32 (21.84–81.88)	
Tumor/tissue ratio of intensity in late phase (170–190 s)			
Mean±SD	0.39±0.1	0.66±0.14	0.000
Median(range)	0.39 (0.21–0.61)	0.66 (0.36–0.94)	

### Arterial phase assessment

Statistical analysis did not reveal significant difference between ICC and HCC in the time of contrast first appearance of tumor, arterial peak maximum time of tumor, and the proportion of homogeneous hyper-enhancement and heterogeneous hyper-enhancement in the arterial phase. The results of software calculation indicated that the peak intensity of tumor and tumor/tissue ratio of intensity were lower in ICC than that in HCC in arterial phase (*p*<0.05).

### Portal phase assessment

**Figure 1:**
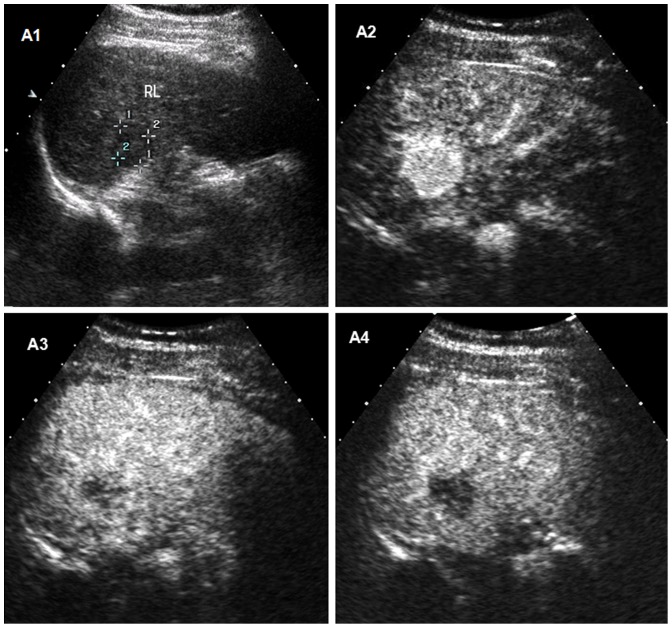
Intrahepatic cholangiocellular carcinoma (ICC) in a 48-year old man with cirrhosis. A1 Conventional ultrasound shows a hypoechoic nodule of 22-enhanced ultrasound, the lesion appears as homogeneous hyperenhancement 19 seconds after the administration of contrast agent. A3 The lesion shows first wash-out 38 seconds after the injection of contrast agent. A4 The lesion appears as marked wash-out at 50 seconds after the administration of contrast agent.

**Figure 2:**
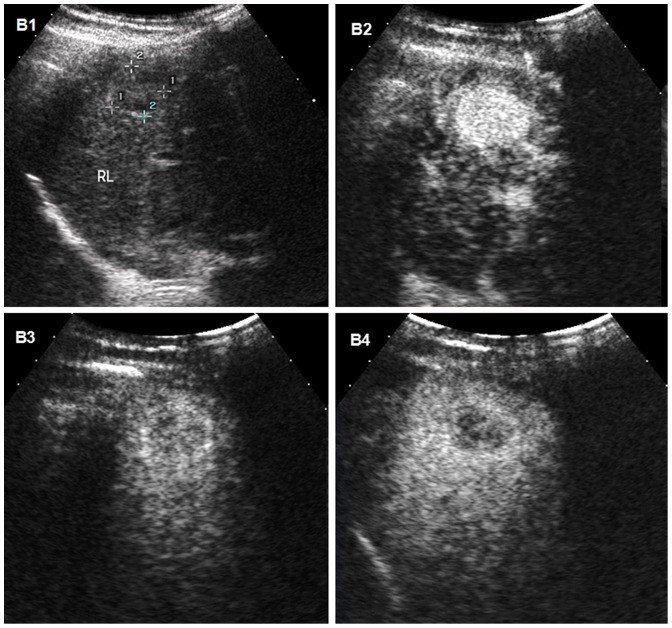
Hepatocellular carcinoma (HCC) with moderate differentiation in a 31-year old man with cirrhosis. B1 Conventional ultrasound displays a hypoechoic nodule of 20-out appearance at 95 seconds after injection of contrast agent. B4 The lesion appears as mild wash-out 179 seconds after the administration of contrast agent.

**Figure 3:**
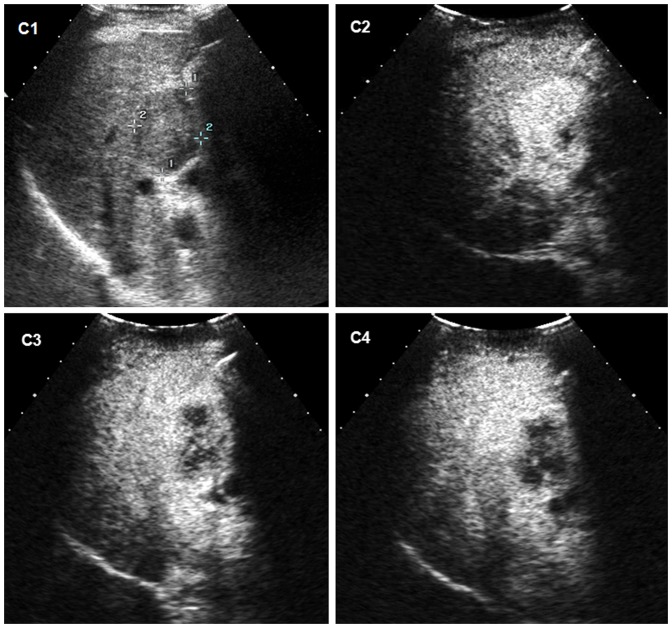
Intrahepatic cholangiocellular carcinoma (ICC) in a 42-year old man with cirrhosis. C1 Conventional ultrasound shows a hypoechoic nodule of 46-out appearance 48 seconds after injection of contrast agent. C4 The lesion appears as marked wash-out 91 seconds after the administration of contrast agent.

**Figure 4:**
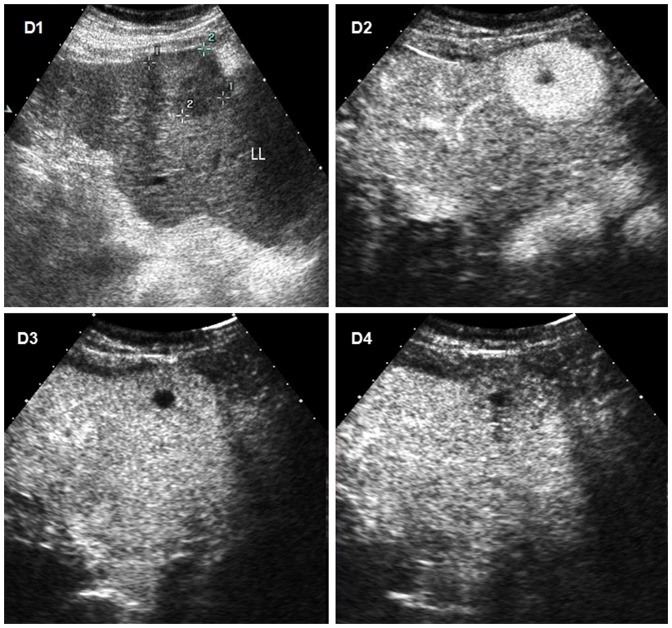
Hepatocellular carcinoma (HCC) with moderate differentiation in a 72-year old man with cirrhosis. D1 Conventional ultrasound shows a hypoechoic nodule of 41(L L) of liver. D2 The lesion shows heterogeneous hyperenhancement 25 seconds after the administration of contrast agent at CEUS, the non-hyperenhancement area at the central part of the lesion indicating necrosis. D3 The lesion shows no wash-out appearance 60 second after injection of contrast agent. D4 The lesion displays mild wash-out 185 seconds after the administration of contrast agent.

**Figure 5:**
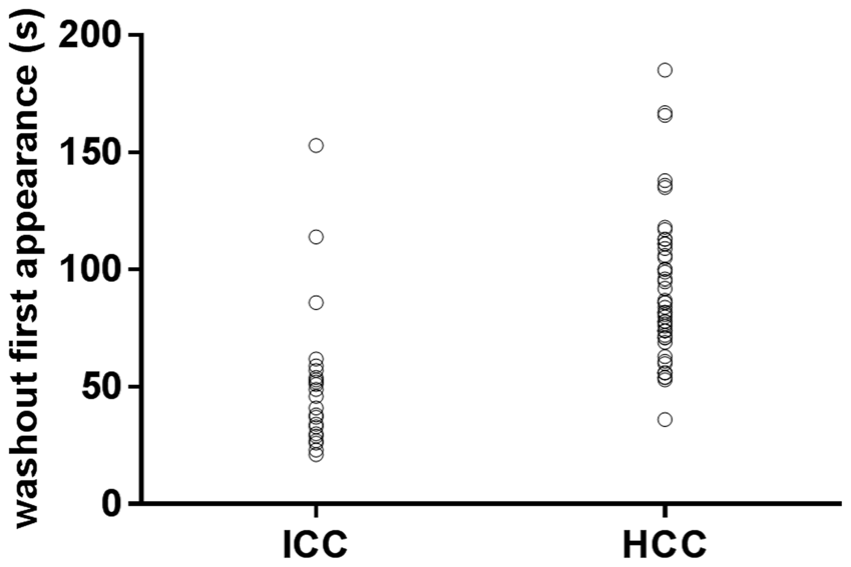
Frequency distribution of washout onset in portal phase (seconds). Washout onset is earlier in patients with intrahepatic cholangiocellular carcinoma (ICC) than in those with hepatocellular carcinoma (HCC).

**Figure 6:**
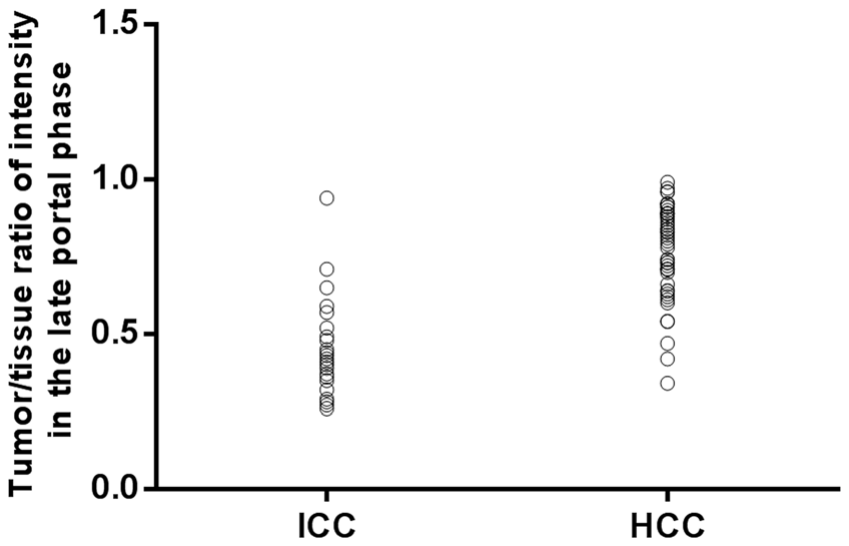
Frequency distribution of peak extent of washout in portal phase (Tumor/tissue ratio of intensity) in portal phase. The peak extent of washout is greater in patients with intrahepatic cholangiocellular carcinoma (ICC) than in those with hepatocellular carcinoma (HCC).

ICC displayed much earlier washout than that of HCC (47.9±26.45 seconds vs 90.9±31.26 seconds) in portal phase, and most ICC (87.9%) showed washout within 60 seconds post contrast agent injection (Fig 1) than HCC did while much more HCC (84.0%) revealed washout after 60 seconds (Fig 2). Under visual assessment by the operator, much more ICC (78.8%) revealed marked washout (Fig 3) than HCC (12.0%) while most HCC showed mild washout or no washout (Fig 4) in late part of the portal phase (90–120 seconds after contrast agent injection). In addition, the results of software calculation indicated that the extent of washout of tumor and tumor/tissue ratio of intensity were lower in ICC than that in HCC during late part of the portal phase (*p*<0.05). The onset of washout and peak extent of washout (tumor/tissue ratio of intensity) in portal phases were demonstrated in plotted graphs (Fig 5, Fig 6). Measurement of tumor/tissue ratio of intensity rather than tumor intensity only was adopted to reflect peak extent of washout in the portal phase because washout was defined as when the lesion displays lower echogenicity than the surrounding liver parenchyma in the portal or late phases preceded by hyper-enhancement in the arterial phase.

Twenty six out of thirty three ICC (78.8%) demonstrated both early washout (<60seconds) and marked washout in late part of the portal phase, whereas, only six of fifty HCC (12.0%) showed this temporal enhancement features (*p* = 0.000).

### Late phase assessment

Marked washout was displayed in more ICC nodules than in HCC nodules (90.9% vs 44.0%) while more HCC nodules showed mild washout or no washout than ICC nodules did in the late phase (56.0% vs 9.1%). The results of software calculation demonstrated that the extent of washout of tumor and tumor/tissue ratio of intensity were lower in ICC than that in HCC during the late phase (*p*<0.01).

Typical dynamic vascular pattern for ICC on CEUS was considered both early washout(<60seconds) and marked washout during late part of the portal phase (90–120 seconds after contrast agent injection) in this study. When this criterion was applied, the diagnostic sensitivity, specificity, positive predictive value, negative predictive value and accuracy were 78.8%,88.0%,81.3%,86.3%,and 84.3% respectively by CEUS.

Of the ICC group, 31 cases had contras-enhanced CT scan and only 1case had MRI scan. Of the HCC group, 48 cases received contras-enhanced CT scan and no patient had MRI scan. Analysis of the dynamic enhancement pattern throughout the different vascular phases in 31 ICC patients showed 14 ICC (45.2%) had a progressive enhancement pattern, 6 ICC (19.4%) stable enhancement pattern, 9 ICC (29.0%) washout enhancement pattern, and 2 ICC (6.5%)other enhancement pattern (one ICC showed peripheral hyperdence in both arterial and portal phases followed by isodense in the delayed phase,the other ICC had isodense in the arterial phase, peripheral hyperdence in the portal phase and followed by hypodence in the delayed phase). The dynamic enhancement pattern throughout the different vascular phases in 48 HCC patients were as follow: 3 HCC (6.3%) had progressive enhancement pattern, 6 HCC (12.5%) stable enhancement pattern, 38 HCC (79.2%) washout enhancement pattern, and1 HCC (2.1%) other enhancement pattern (global hyperdence in both arterial and portal phases followed by isodense in the delayed phase).

Typical dynamic vascular pattern for ICC on contrast enhanced CT was considered either progressive enhancement pattern or stable enhancement pattern [14].When this criterion was adopted, the diagnostic sensitivity, specificity, positive predictive value, negative predictive value and accuracy were 64.5%,81.3%,69.0%,78.0%,and 74.7% respectively by contrast enhanced CT.

Statistical analysis showed no difference in the diagnostic sensitivity, specificity, positive predictive value, negative predictive value and accuracy between CEUS and contrast enhanced CT (*p*>0.05).

## Discussion

CEUS has been introduced about 10 years ago for characterization of focal liver lesions, and its excellent diagnostic value established by many prospective studies including the German DEGUM-study with over 1000 patients [15] and the French multicentre study [16]. CEUS was proven able to characterize liver tumors with at least the same accuracy range as contrast enhanced computed tomography and contrast enhanced magnetic resonance imaging [17], [18]. Therefore, CEUS has been included together with CT and MRI in the imaging techniques accepted for the non-invasive diagnosis of HCC in the guidelines from the American Association for the Study of Liver Diseases (AASLD) 2005[4]. However, in a retrospective series of 21 patients with histologically confirmed ICC on cirrhosis collected between 2003 and 2009, the Barcelona Clinic Liver Cancer (BCLC) group found that ten ICC had similar CEUS enhancement pattern considered diagnostic for HCC, consisting in homogeneous arterial hyper-enhancement followed by washout [9].Based on the results of this study, CEUS has been dropped from the diagnostic techniques of nodules in cirrhosis in the updated AASLD guidelines 2011[19] and subsequently also from the guidelines of European Association for the study of the liver (EASL) [5].However, this removal has been raised much controversial arguments and was not well received in Europe and Asia[12].

The results of our study revealed arterial peak intensity of tumor and tumor/tissue ratio of intensity in the arterial phase were lower in ICC than that of HCC, and this was in agreement with a previous study [20].Our study demonstrated that 69.7% of ICC with heterogeneous hyper-enhancement and 30.3% of ICC with homogeneous hyper-enhancement in the arterial phase and 88% of ICC had early wash out in 60 seconds. The rate of ICC showing homogeneous hyper-enhancement in the arterial phase is lower than those of two previous studies (47.6% and 52% respectively) by Vilana et al [9] and Galassi [11].More ICC demonstrated early washout in our study than those reported by Vilana et al [9] which showed that 29% of ICC had washout beyond 120 seconds. An explanation of this difference could be the tumor size, which was larger in our study (median size of the tumors was 40 mm) as compared with the other two studies (median tumor size was 32 mm and 29 mm respectively).Another possible reason could be the etiology of cirrhosis. In the present study, 28 of the 33 (84.8%) patients with ICC were positive for HBsAg. In contrast, most patients with ICC (62.5% and 71.4% respectively) in cirrhotic liver were related to HCV infection in the two previous studies [9], [11].The rate of hepatitis B infection in ICC of our study is really much higher than other countries. Actually, most HCC occur in patients with cirrhosis in Asia, especially in China. Patients with cirrhosis who undergo surveillance may have an earlier stage of HCC at diagnosis [21]. Unfortunately, surveillance for HCC in patients with cirrhosis is recommended but may not be successfully performed. Less than 20% of patients with cirrhosis who developed HCC received regular surveillance in the United States [22].HCC were detected during surveillance in the minority of patients even at major referral center[23].Surveillance for HCC is still not a consolidated practice as it should be[24]. Previous study also demonstrated that more than half of ICC (11/21, 52.4%) in cirrhosis were larger than 30 mm in size, and the largest one reached 70 mm [9].Therefore, differential diagnosis of large ICC from large HCC is still a more common clinical situation worldwide for the majority of liver tumors are found not at very early stage in cirrhotic patients. It is necessary to point out that large nodules showing heterogeneous hyper-enhancement in arterial phase followed by washout in the portal or late venous phase are also liable to the diagnosis of HCC, for CEUS definition of HCC was complete hypervascularity or partial hypervascularity (due to non-enhancing areas indicating necrosis) in arterial phase with a chaotic and irregular vascularisation followed by hypo-enhancing pattern in portal or late phase [8]. Therefore, typical enhancement pattern for HCC should not be confined to global hyper-enhancement in arterial phase followed by washout in the portal or late venous phase.

The present study demonstrated that most ICC (87.9%) showed washout before 60 seconds than HCC did (16.0%).Vilana et al also reported that half of ICC in cirrhosis showed washout before 60 seconds at CEUS [9]. Galassi et al found that more than half of ICC showed washout during portal phase though it was not specified the exact timing of first occurrence of washout [11].On the other hand, Boozari et al [25] reported in a prospective study in cirrhotic patients that even moderately or poorly differentiated HCC had a rather late onset of washout (120±71.8 seconds) and it is longer in well differentiated HCC (164±2.1seconds),indicating most HCC manifest much slower washout appearance in cirrhosis.

In this study, we found that much more ICC (78.8%) displayed marked washout than HCC did while most HCC (88%) showed mild washout or no washout in late part of the portal phase. Mild or moderate wash-out is considering as typical for HCC by the recent release of the EFSUMB liver CEUS guidelines [26].

Further analysis of our data revealed that most ICC (78.8%) demonstrated both early washout(<60seconds) and marked washout during late part of the portal phase, whereas, only few cases of HCC (12.0%) showed this temporal enhancement features. When these dynamic CEUS features were applied in differentiating ICC and HCC in cirrhosis, CEUS has similar diagnostic efficacy to contrast enhanced multi-detector-row CT. The possibility to grade the intensity of the wash-out in the late venous phase applies to all contrast imaging techniques (including CEUS,CT and MRI), whereas the possibility to establish an accurate temporal pattern of enhancement belongs to CEUS only, due to it's a real time imaging modality [26], [27], [28].

Therefore, our data are satisfactory to state that when a nodule displayed early washout (namely before 60 seconds post contrast agent injection) and marked washout in late part of the portal phase at CEUS in cirrhosis, it is no longer typical for HCC, but highly suspicious of ICC.

There were some limitations to our study. First, it was limited by its retrospective nature though we provide a relatively large series of pathologically proven ICC in cirrhosis seen at CEUS and contrast-enhanced multiphasic multi-detector CT. Our results need to be validated in prospective studies in the future though it is difficult because the low incidence of ICC in cirrhosis. Second, it is impossible for us to compare the diagnostic efficacy of CEUS and MRI because only one case out of 33 cases of ICC had MRI scan in our series, and this reflects the fact that in many countries worldwide, with a high incidence of HCC, the availability of MRI is very low.

In conclusion, the data of our study demonstrated that analysis of detailed temporal enhancement features on CEUS may help differentiate ICC from HCC in cirrhosis.If a nodule in cirrhotic liver displays hyper-enhancement in the arterial phase followed by early and marked washout in the portal phase, the nodule is highly suspicious of ICC rather than HCC. when considering a refinement of CEUS diagnosis by more detailed temporal features, namely, timing of washout emergence and degree of intensity of washout in the portal phase, the proportion of ICC cases at risk of misdiagnosis with HCC would decreased in cirrhotic patients.
